# Intracellular redistribution of neuronal peroxisomes in response to ACBD5 expression

**DOI:** 10.1371/journal.pone.0209507

**Published:** 2018-12-27

**Authors:** Yunhong Wang, Jeremy Metz, Joseph L. Costello, Josiah Passmore, Michael Schrader, Christian Schultz, Markus Islinger

**Affiliations:** 1 Institute of Neuroanatomy, Center for Biomedicine & Medical Technology Mannheim, Medical Faculty Mannheim, University of Heidelberg, Mannheim, Germany; 2 Biosciences, University of Exeter, Exeter, United Kingdom; Weizmann Institute of science, ISRAEL

## Abstract

Peroxisomes can be frequently found in proximity to other subcellular organelles such as the endoplasmic reticulum (ER), mitochondria or lysosomes. The tail-anchored protein ACBD5 was recently identified as part of a tethering complex at peroxisome–ER contact sites, interacting with the ER resident protein VAPB. Contact site disruption was found to significantly increase peroxisome motility, apparently interfering with intracellular positioning systems. Unlike other somatic cells, neurons have to distribute organelles across relatively long distances in order to maintain their extraordinary cellular polarity. Using confocal live imaging microscopy in cultured hippocampal neurons we observed that peroxisomes and mitochondria show a strikingly similar motility with approximately 10% performing microtubule-driven long range movements. In order to investigate if ER contacts influence overall peroxisome motility and cellular distribution patterns, hippocampal neurons were transfected with plasmids encoding ACBD5 to stimulate peroxisome–ER interactions. Overexpression of ACBD5 reduced peroxisomal long range movements in the neurites of the hippocampal cells by 70%, implying that ER attachment counteracts microtubule-driven peroxisome transport, while mitochondrial motility was unaffected. Moreover, the analyses of peroxisome distribution in fixed neurons unveiled a significant redistribution of peroxisomes towards the periphery of the perikaryon underneath the plasma membrane and into neurites, where peroxisomes are frequently found in close proximity to mitochondria. Surprisingly, further analysis of peroxisome and VAPB distribution upon ACBD5 expression did not reveal a substantial colocalization, implying this effect may be independent of VAPB. In line with these findings, expression of an ACBD5 variant unable to bind to VAPB still altered the localization of peroxisomes in the same way as the wild-type ACBD5. Thus, we conclude, that the VAPB-ACBD5 facilitated peroxisome-ER interaction is not responsible for the observed organelle redistribution in neurons. Rather, we suggest that additional ACBD5-binding proteins in neurons may tether peroxisomes to contact sites at or near the plasma membrane of neurons.

## Introduction

In order to maintain their extraordinary cellular asymmetry, neurons have to closely coordinate transport processes into dendritic and axonal compartments. To this end, long distance trafficking and local distribution of organelles are determined via microtubule polarity along axons and dendrites [1]. Furthermore, organelles have to be positioned at sites of specific physiological demands. Mitochondria, for example, have to be precisely immobilized at energy-demanding cellular regions such as synapses [2]. For such intracellular positioning, the organelles require specific docking proteins. One example is syntaphilin, which has been recently reported to reside on axonal mitochondria and work as a stationary docking factor, immobilizing the organelles on the microtubule cytoskeleton [3]. Currently, it is becoming increasingly obvious that different organelles, such as mitochondria and the endoplasmic reticulum (ER), in addition to their relationship with cytoskeletal elements, maintain interorganellar contact sites which might influence dynamic transport processes. Interestingly, Lippincott-Schwartz and coworkers observed that a functional microtubule cytoskeleton appears to generally influence organelle contact site formation, implying a causal relationship between organelle motility and contact site formation [4].

Peroxisomes (PO) are ubiquitous organelles with crucial roles in the metabolism of lipids and reactive oxygen species (ROS) [5]. Their importance for the brain is highlighted by the existence of various inherited PO disorders which usually exhibit a severe neurological pathology [6]. Analysis of conditional Pex5 knockout mice, which lack POs in oligodendrocytes or neurons, respectively, showed that while the absence of PO from oligodendrocytes inflicted the most severe brain pathology, PO inside projection neurons appear to play a more subtle role in neuronal physiology [7]. Nevertheless, a functional neuronal peroxisomal β-oxidation has been recently shown to be required for axonal integrity of Purkinje cells [8] and peroxisomal ROS metabolism was reported to influence activities of pro-opiomelanocortin producing neurons in the hypothalamus [9] underlining the physiological importance of POs in neurons. In most cells, POs appear to be evenly distributed in the cytosol [10]. In contrast, neuronal POs preferentially accumulate in axon terminals during early postnatal development [11] but are virtually absent from the axon in mature projection neurons [12]. Hence, regulated trafficking processes appear to be required to maintain the asymmetric peroxisomal distribution in neurons.

As observed in other cell types, POs in neurons perform microtubule-dependent long range movements [13, 14]. Another feature of POs in numerous cell types is that they are regularly found in close contact with the ER [15]. We and others recently showed that physical PO-ER interactions can be facilitated by a molecular tether between the two tail-anchored proteins ACBD5 and VAPB, which reside on the peroxisomal and ER membrane, respectively [16, 17]. Moreover, knockdown of ACBD5 was not only found to reduce PO-ER interactions but also to significantly increase PO motility in cultured human fibroblasts [16, 17]. As mutations in both proteins are disease relevant–causing a variant of familial ALS [18] and the PO disorder ACBD5-deficiency [19]–and result in a severe neurologic pathology, we aimed at analyzing the significance of their molecular interaction in neurons. Specifically, we asked if a change in ACBD5 expression might alter motility and, as a consequence, distribution of POs in the highly polarized neurons. To clarify this issue, we overexpressed ACBD5 in mouse hippocampal primary cultures and measured peroxisomal movements and abundance in the neurites. Interestingly, peroxisomal long range movements were largely diminished in response to ACBD5 overexpression. Moreover, PO localization significantly changed in ACBD5-transfected neurons: compared to control neurons POs were found to preferentially accumulate along the plasma membrane. In addition, PO numbers in neurites increased, while PO density in the soma was decreased. Unexpectedly, the alterations in PO motility and distribution in the hippocampal neurons were independent of the interaction between ACBD5 and VAPB. We conclude that ACBD5 interacts with additional tethering proteins in neuronal cells eventually leading to PO redistribution.

## Materials and methods

### Plasmids and antibodies

All plasmids were cloned and amplified using DH5α Competent Cells (Invitrogen, Cat. No. 18265–017). The plasmids encoding EGFP-SKL, myc-ACBD5(human), FLAG-ACBD5(human)-FFAT, ACBD5(human)-ACB and FLAG-ACBD5(human)-WT were generated as described in a previous publication (16). The plasmids mCherry2-C1, mPlum-N1, mPlum-Mito-3 and mCherry-Mito-7 were gifts from Michael Davidson (Addgene plasmid #54563, #54629, #55988, #55102) [20]. The primary and secondary antibodies used in this study include mouse monoclonal anti-GFP (MAB3580, Chemicon), mouse monoclonal anti-myc (#2276, Cell signaling), rat monoclonal anti-RFP (5F8, Chromotek), rabbit polyclonal Pex14 (kind gift from D. Crane, Brisbane, Australia), rabbit polyclonal ACBD5(human) (HPA012145, Sigma), rabbit polyclonal VAPB (HPA013144, Sigma), Alexa Fluor 488 anti-mouse IgG, Alexa Fluor 568 anti-rat IgG and Alexa Fluor 647 anti-rabbit (A32723, A11036, A32733, Invitrogen).

### Cell culture and transfection

Primary hippocampal neuron cultures were prepared from E18 embryonic C57/BL6J mice using a previously described protocol [21] with slight modifications. Pregnant C57BL/6 mice were purchased and delivered at E11 (Janvier, Labs, France) and kept in the in-house animal facility for one week till the culture preparation day. All animals had access to food and water *ad libitum* and were housed in a standard 12/12 h light/dark cycle. Animal protocols were approved by the Ruhr-University Animal Research Board and the State of Baden-Württemberg. Briefly, the dissected hippocampi were collected from sacrificed E18 embryos and digested by papain enzyme (100 units per ml, Worthington, Biochemical Corp.) for 20 min at 37°C. Later the digested hippocampi were triturated and filtered to remove tissue aggregates in order to obtain a homogenous cell suspension. Subsequently, the cells were separated from debris using a centrifugation in BSA solution (7.5%, PAN, BIOTECH) for 5 min at 1000 rpm. After centrifugation cellular debris floated on top of the BSA solution while preserved cells were collected as a pellet and resuspended. Dissociated cells were then seeded into either coated 24-well plates (Sarstedt) or 35mm imaging dishes (ibidi) with a density of ~12000 cells/cm^2^ using Neurobasal medium(Gibco, Life Technologies) supplemented with B27 (1%, Gibco, Life Technologies), horse serum (1%), Glutamax (1%, Gibco, Life Technologies), penicillin/streptomycin (10,000 units/ml, 0.5%). To maintain the culture, half of the medium was replaced 2 or 3 times a week by medium containing B27 (2%), Glutamax (1%) and penicillin/streptomycin (10,000 units/ml, 0.5%). The cultures were transfected using the calcium phosphate precipitation method at DIV 7 to 9 as described before [22] with slight modifications regarding the reaction volume. 27.5μl of reaction volume containing: DNA (1.5~3μg, see below), CaCl_2_ (125mM), HBS (137 mM NaCl, 4.75 mM KCl, 7.5 mM glucose, 21 mM HEPES, 0.7 mM Na_2_HPO_4_, pH 7.05) was added into each 500 μl growth medium of neuronal culture. For the live cell imaging approach the following amounts of plasmid DNA were added per transfection reaction: 1 μg EGFP-SKL + 1 μg of mCherry-Mito-7/mPlum-Mito-3 or 1 μg myc-ACBD5 + 1 μg EGFP-SKL + 1 μg of mCherry-Mito-7/mPlum-Mito-3, respectively. For the end point measurement of the PO distribution, transfections were performed with the following plasmid DNA amounts: 0.75 μg EGFP-SKL + 0.75 μg of mCherry2-C1/mPlum-N1 or 0.75 μg of myc-ACBD5/FLAG-ACBD5-FFAT/FLAG-ACBD5-ACB/FLAG-ACBD5-WT + 0.75 μg of mCherry2-C1/mPlum-N1. After 1 hour of incubation at 37°C, the transfection mixture was washed out of the culture using pre-warmed HBSS (Gibco, Life technologies) before the culture continued the daily maintaining.

### Live-cell imaging

Neurons cultured in Ø35 mm imaging dishes with polymer coverslip bottom compatible for laser scanning were co-transfected at DIV 7 to 9. Cultures were divided into two groups, one was transfected with fluorescent PO marker plasmid coding for EGFP-SKL, the mitochondria marker mCherry-Mito-7 and with the myc-ACBD5 encoding plasmid while the second group was transfected without the latter plasmid. Because it was not possible to visualize the expression of myc-ACBD5 under live conditions, a pre-experiment to evaluate the efficiency of the co-transfection of myc-ACBD5 with the other two plasmids was conducted. Immunostaining by anti-myc, anti-GFP and anti-RFP on the fixed culture 24 hours after transfection with the 3 kinds of plasmids was performed. Co-transfection efficiency of EGFP-SKL, mCherry-Mito-7 and myc-ACBD5 constructs was 80~90% of transfected neurons expressing all 3 kinds of plasmids. For each Ø35 mm imaging dish 55μl of transfection mixture was used containing the different DNAs with equal ratio. After 24 hours of expression, neurons were imaged using Leica SP5 inverted confocal microscope equipped with 63×/1.4 oil objective (Leica) and resonance scanner to provide a scanning speed up to 8000 Hz. Physiological environment was also controlled with temperature 37°C, gas: 95% O_2_, 5% CO_2_, and humidity supplied. Argon 488 nm and He 561 nm laser lines were used during the imaging session. Time-lapse images focused on each individual transfected neuron with two channels were simultaneously acquired every 4~5 seconds with 1024*1024 pixels, z-stacks of about 4~6μm with 0.4μm step size, and a total imaging time for about 8 minutes.

### Immunofluorescence and microscopy

Neuronal cultures grown on glass coverslips were fixed 24 hours after transfection by 4% paraformaldehyde (PFA) in PBS (pH 7.4) for 10 minutes, followed by a 1 hour combined blocking and permeabilization step (1% BSA, 0.2% fish skin gelatin, 0.1% Triton X-100). Cells were then incubated with primary antibodies overnight and secondary antibodies for another 1 hour. The coverslips with the stained neuronal cultures were shortly rinsed in ddH_2_O and then mounted upside down onto glass slides with immersion medium (Roti, FluorCare). All immunofluorescence staining procedures were performed at room temperature and with 3 times of PBS rinsing of the coverslips between each antibody incubation step. Confocal images were collected using a Nikon 90i upright microscope mounted with Plan Apo 100×/1.45 NA oil objective (Nikon). Argon 488 nm and He 543 nm 633nm laser lines were used. Each image was focused on single neuron in the center which exhibited good signal to noise ratios for all channels. Imaging parameters comprise 1024*1024 pixels, fixed 0.08μm pixel size, z-stacks with 4~7 μm depth and 0.3 μm scanning thickness.

### Peroxisome distribution measurements

Neurons co-expressing EGFP-SKL or myc-ACBD5 as peroxisome markers and cytoplasmic mCherry (mCherry2-C1) used to track the neuron morphology were immunostained with anti-GFP, anti-myc, and anti-RFP antibodies, respectively. Moreover, to visualize the total POs in the culture as a positive control, anti-Pex14 as a generally applied peroxisomal marker was used to complete a triple-labeling of the culture. After the confocal imaging, the collected images including 59 imaged pyramidal-type neurons for the EGFP-SKL control group and 77 for myc-ACBD5 group were pre-processed including brightness/contrast adjustment and background subtraction to facilitate the upcoming channel segmentation by Fiji (ImageJ, National Institutes of Health). The neuron soma area was first delineated manually and then subtracted from the thresholded and segmented mCherry channel which defined the focused neuron area to obtain the neurite area outline from the image. Subsequently, the segmented peroxisome particles were determined within the outlined soma and neurite area respectively. A semi-automatic user-defined plugin written by ImageJ macro language was used to implement all above mentioned analysis procedures. The number and area of peroxisomes of each imaged neuron including soma and neurite area were for comparison documented separately for the two groups.

### Peroxisome and mitochondria motility measurements

After 24 hours of expression, two cell culture groups that were co-transfected with the plasmids encoding EGFP-SKL and mCherry-Mito-7 with and without myc-ACBD5 were imaged under live conditions. Neurons that co-expressed EGFP-SKL and mCherry-Mito-7 emitting the fluorescence with acceptable intensity were selected for time-lapse imaging. A total of 20 neurons were collected for each group. The images were then brightness/contrast adjusted manually by Fiji image software. The neurites where the POs located relatively sparsely were preferentially used to generate kymographs for the PO motility analysis. The trajectories of the detected PO and mitochondrial movements were obtained first by adding up all the frames of each time-lapse image, segmented line tools were used to draw lines along the neurites to cover the organelle trajectories. The line width was adjusted to cover the maximum size of the organelle in the neurite of interest. Next, kymographs were generated from the line-selected neurites by kymograph plugin using Fiji (ImageJ, written by J. Rietdorf and A. Seitz). After image enhancing and inversion of the kymograph, the segmented line tool was used to draw lines along the intensity lines, which represent the excursions of either static or moving POs and mitochondria in the time space. Velocities and travelled distances of the captured organelles were read out through these lines by using a slightly modified version of the ImageJ “velocities” macro from the plugin above.

The contacts of POs and mitochondria were counted manually only in the neurite area by using a similar method as described for PO distribution analysis. All the first frames of each time-lapse image were collected for the analysis. First, the soma area of each neuron was delineated and removed from both PO and mitochondria channels. Then the two channels indexed by distinct colors were merged after thresholding with the Otsu method and binarizing. The total number of POs and those POs that are free from mitochondria were measured respectively. The compiled data, absolute number of POs that contact mitochondria, % of PO-mitochondria contacts, were then used for the comparison between the two groups.

To generate more objective quantitative data on the PO statistics and mitochondria motility analysis, we used a custom image analysis pipeline written in Python. In brief, neurons were segmented from the maximum projection of both channels (PO and mitochondria) and all time-points of data. This was done by first running a multi-scale Frangi ridge filter [23] over the image, followed by non-maximal suppression, double threshold, and hysteresis tracking; in this way our neurite tracing algorithm can be seen as a modification to the standard Canny edge detection algorithm [24], adapted to detect ridges. Once the neurites have been detected using the aforementioned approach, a scale-space Difference of Gaussian filter [25] was applied to detect the soma, identified as the largest detected object, was subtracted from the neurite mask to avoid detecting POs in the soma region. Using the neurite masks obtained from the neuron segmentation, detection of the organelles was a greatly simplified task, and was achieved by blurring each frame with a Gaussian filter (sigmas 1 and 2 for POs and mitochondria respectively). Next, an automated threshold was calculated using Otsu's algorithm applied to only filtered pixel values with the neurite mask region. This threshold was used to binarize the image into organelle and non-organelle regions. Lastly, these regions were grouped using a connected component labelling algorithm. The detected organelle regions were then tracked using a global nearest neighbors algorithm applied to centroid positions, which links nearby objects in adjacent frames in a globally optimal manner. These linked object positions, or trajectories, were the basis of all subsequent analysis.

Each PO’s and mitochondria’s maximum and average speeds (μm/s), total travelled distance (μm), as well as the net travelled distance (μm) were collected for each group and compared between the two groups. Furthermore, the movements of POs and mitochondria were assigned to four categories according to the different travelled distances over the entire imaging session of each organelle: static (< 1 μm), very short range movement (1 μm– 5 μm), short range movement (5μm– 10μm), long range movement (≥ 10 μm). The percentages of POs and mitochondria with different types of movement were compared as well.

### Statistical analyses

To determine the statistical difference between the ACBD5-transfected and control group, a one-tailed, unpaired t-test was used (*P < 0.05; **P < 0.01; ***P < 0.005; ****P < 0.001; ns: not significant) by SigmaPlot (Systat Software Inc.). Sample sizes of different kinds of data were indicated under the corresponding data collecting methods. All the quantitative data were collected from at least three independent experiments and presented as mean ± SEM.

## Results

### Peroxisomes are in close proximity to the ER and mitochondria in neurites of hippocampal neurons

**Fig 1:**
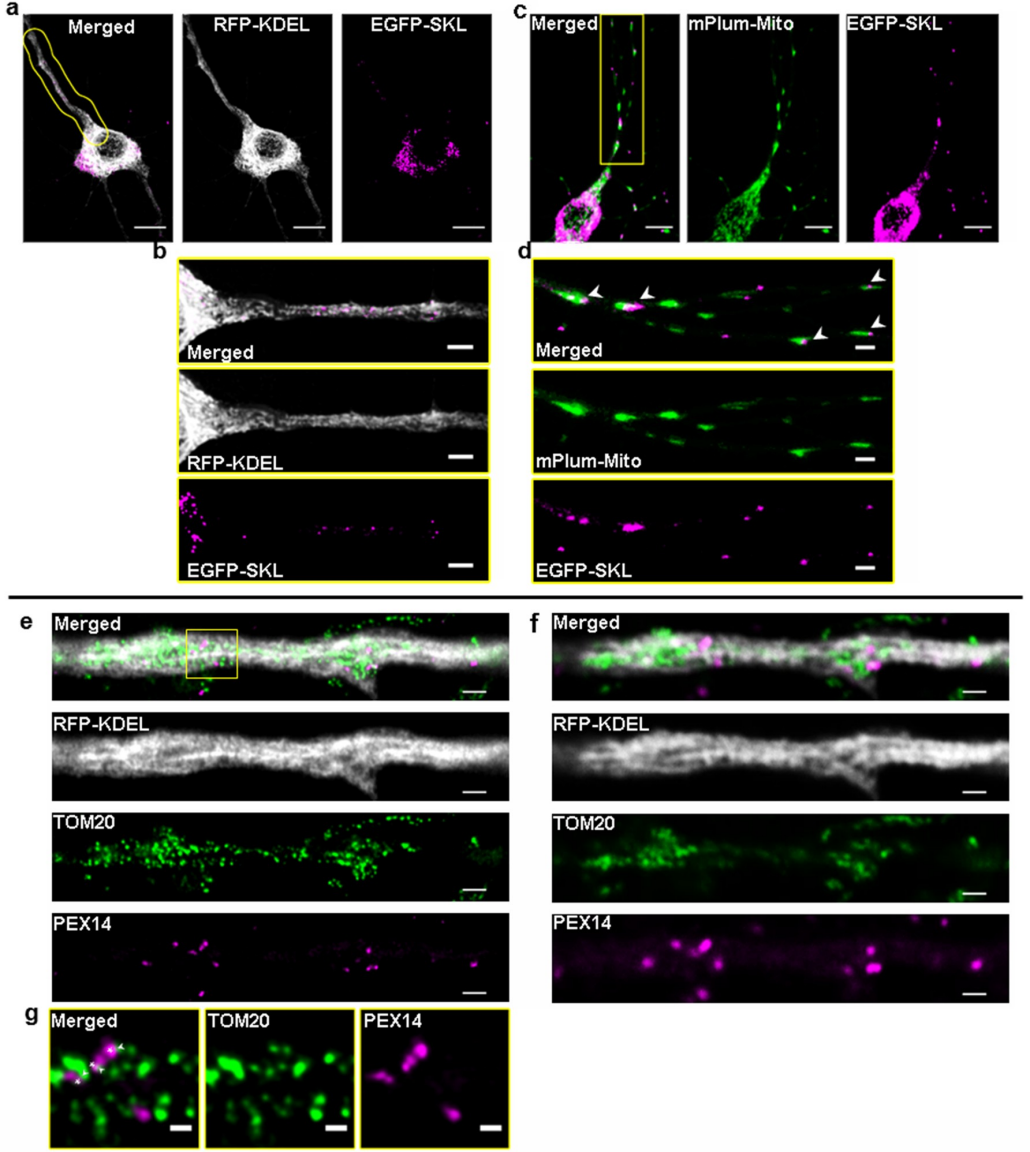
Visualization of PO, mitochondria and ER distribution in hippocampal neurons using confocal and STED microscopy. (a) Overview of a neuron with POs and ER marked by expression of EGFP-SKL (magenta) and RFP-KDEL (grey), respectively using standard confocal microscopy. (c) Overview of a neuron with POs and mitochondria marked by expression of EGFP-SKL (magenta) and mPlum-Mito-3 (green) [30], respectively using live confocal microscopy, shown as maximum intensity projection. Scale bars comprise 10 μm. (b, d) Magnifications of the areas marked by yellow lines in a, c. Note that POs often appear in close vicinity to the ER and mitochondria (Scale bars: 3 μm). (e) STED and (f) confocal image of the same neuron showing peroxisomal Pex14 (magenta) and mitochondrial TOM20 (green) (30) antibody staining. The ER (grey) was marked by expression of a RFP-KDEL encoding plasmid (Scale bars: 1μm). (g) Magnification of the area marked by a rectangle in (e), maximum pixel resolution in the image is 20 nm, thus signals attached to each other are in the distance characteristic for organelle contact zones (Scale bar: 300nm).

POs share an intricate relationship with the ER as well as mitochondria, which is morphologically documented by close intracellular proximities, implying the existence of specific contact sites between the different organelles [26, 27]. A recent study in COS-7 cells using simultaneously expressed marker fluorescent proteins for six subcellular compartments reported that > 90% of POs are in contact with the ER and around 20% in contact with mitochondria [4]. Neurons with their long dendritic and axonal extensions exhibit a highly specialized morphology and cellular polarity which requires a tight organization of the intracellular distribution and interaction of their main subcellular compartments. To evaluate how POs in neurons distribute in relation to their major organellar interaction partners, hippocampal neurons were transfected with plasmids expressing EGFP-SKL-PO, RFP-KDEL-ER, and mPlum-Mito-3 to serve as PO, ER and mitochondrial marker proteins, respectively. The high organelle density in the somatic area of the neurons significantly impedes light microscopic analysis of organelle contacts whereas the more flattened and extended morphology of the dendrites allowed us to more clearly resolve individual organelles (Fig 1A). As depicted in Fig 1B (enlarged and straightened outlined area in Fig 1A), POs are regularly found embedded in a network of ER tubules in a confined space of dendritic processes, making it impossible to distinguish between real organelle associations and arbitrary overlap of fluorescent signals. Interestingly, an unexpectedly high proportion of POs was found in close proximity to mitochondria in the dendrites (overview in Fig 1C, arrowheads in Fig 1D,). Such proximities might mirror the functional relationship between both organelles, which exchange fatty acids generated in β-oxidation and cooperate in the metabolism of ROS [28]. Since the resolution of standard confocal microscopy (Fig 1F) does not allow us to distinguish if these organelle proximities might be close enough to depict organelle contact sites (< 30 nm), we applied STED microscopy (with a maximum resolution of 20 nm/pixel) using comparable sets of antibodies and marker plasmids. Unlike in standard confocal microscopy, with STED microscopy the fluorescent signals for the antibodies against Pex14 (subunit of PO matrix protein import complex) and TOM20 (subunit of the mitochondrial outer membrane import receptor) do not represent whole organelle structures but mirror the macromolecular organization of the organelle membranes. In this respect, Pex14 and TOM20 fluorescent signals do not necessarily show individual organelles but instead show the respective protein import complexes on the membrane of different round or elongated organelle structures whilst the soluble ER marker RFP-KDEL distributed more homogenously inside the ER network (Fig 1E). Importantly, signals for Pex14 and TOM20 can be found in close proximities in the 20–40 nm range (overview in Fig 1E, enlarged in Fig 1G, arrowheads and asterisks), which would position respective PO and mitochondrial membranes at a distance which is characteristic for organelle contact sites [29]. Similarly, the dense ER network in the dendrites of hippocampal neurons tightly surrounds the Pex14 and TOM20 signals, implying a physical relationship between the organelles. According to these observations, correlating STED and confocal microscopy, we concluded that the neurite regions of the hippocampal neurons not only allow analysis of kinetic and static PO rearrangements but also permit monitoring of their interaction with mitochondria and the ER. We therefore focused on the dendritic compartment of the hippocampal neurons, to investigate if expression of molecular linker structures, i.e. the ACBD5-mediated PO-ER tether, alters organelle interaction/communication networks in neuronal cells.

### Peroxisomes and mitochondria exhibit comparable motilities in hippocampal neurons

**Fig 2:**
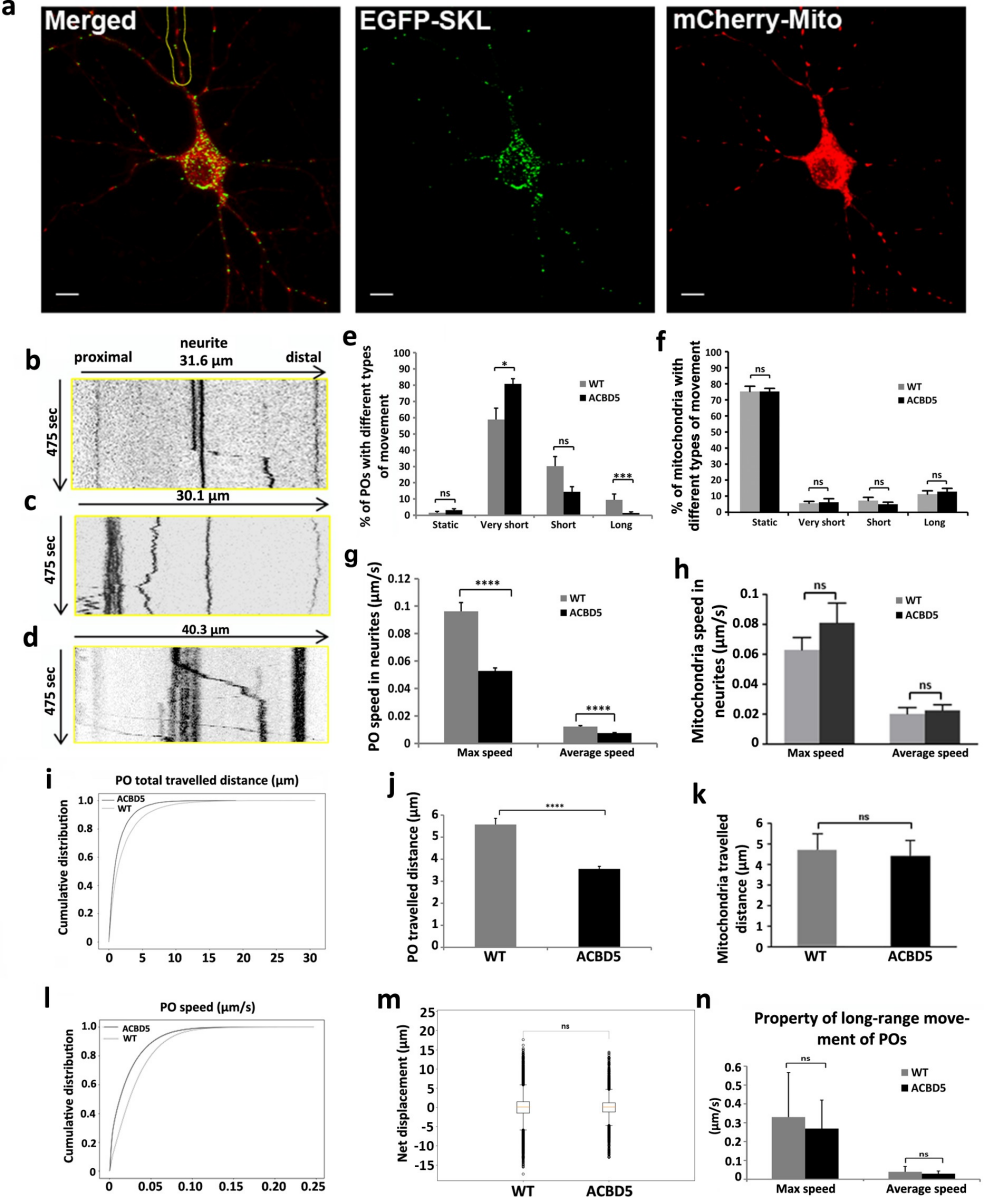
Live confocal imaging analysis of POs and mitochondria in hippocampal neurons. (a) POs and mitochondria were identified by expression of EGFP-SKL and mCherry-mito-7, respectively (Scale bar: 10 μm). Images are displayed as maximum intensity projections. (b-d) Representative kymographs illustrating peroxisomal (b, c) and mitochondrial (d) movements. Kymograph (b) represents the region of the neurite highlighted in (a). The kymographs were generated from the proximal 30~40 μm of neurites, straight vertical lines show static POs and mitochondria; Fig 2B includes a rapid saltatory movement across approximately 10 μm. (e, f) Classification of PO and mitochondria motility into long range movements (> 10 μm), short range movements (5 μm– 10 μm), very short range movements (1 μm– 5 μm), and static organelles (< 1 μm) in myc-ACBD5 expressing and control neurons (total analysis time = 8 min). (g, h) Maximum and average speed (μm/s) of all measured POs and mitochondria in neurites of ACBD5 expressing and control cells. (j, k) Averaged total travelled distance (μm) of measured POs and mitochondria in ACBD5 expressing and control neurons. (i, l) Cumulative distribution of PO travelled distance and speed. (m) Directionality of PO movements in the neurites. Box plots show the PO motility as cumulative net displacement (μm) of anterograde and retrograde movements of all POs in response to ACBD5 expression. Proportions of anterograde (positive values) and retrograde (negative values) movements remain stable after ACBD expression. (n) Comparison of the maximal actual speed and average speed of POs with long range movement between the two groups. Number of organelles analyzed: in control neurons 323 POs, 307 mitochondria; in myc-ACBD5 expressing neurons 360 POs, 267 mitochondria.

Since neurons particularly rely on efficient transport systems to facilitate correct organelle distribution along their extended dendrites and axons, we tested if changes in ACBD5 expression, intended to alter contact site formation, would influence PO motility. To decipher if the expression of ACBD5 changes PO motility we initially characterised movement of POs and mitochondria in untreated hippocampal neurons with a pyramidal morphology (WT neurons). To visualize POs and mitochondria, hippocampal primary cultures were transfected at DIV7~9 with plasmids coding for EGFP-SKL and mitochondria-targeted mCherry (mCherry-Mito-7) (Fig 2A). To analyze velocities and travelled distances of POs and mitochondria, the transfected neurons were recorded for 8 min and individual organelles in the neurites analyzed by kymograph trajectories. As exemplified by representative kymographs (Fig 2B, 2C and 2D), POs generally showed no continuous movements and did not trespass the whole investigated dendrite section in the analyzed time frame. Rather, they generally reside at a fixed position, perform a restricted saltatory movement of several μm and are then again arrested (Fig 2B and 2C). Therefore, an averaged total travelled distance of all the analyzed POs for the whole time of analysis gives a relatively low value of 5.5 μm in the control group (Fig 2J). Compared to POs, mitochondria appear to move more constantly along the neurites, showing frequent fusion and fission events (Fig 2D, S1 Movie). However, a detailed kymograph analysis of mitochondrial movements as exemplified by Fig 2F revealed similar motilities to POs, showing low numbers of movements above a distance of 5 μm. Thus the apparently more dynamic appearance of mitochondria in the supplementary movies (S1 and S2 Movies) is merely caused by their higher total numbers in the neurites. Like POs, mitochondria showed average travelled distances of approx. 5 μm (Fig 2K). As described for other cell types [31], POs in dendrites of EGFP-SKL expressing controls were classified to be either static (movements < 1 μm), perform oscillating (very short) movements (1–5 μm), short range movements of 5–10 μm or long range movements exceeding distances of 10 μm (Fig 2E). With a proportion of 60% (59% of POs exhibiting very short range movement and 1% of static POs), most POs did not show significant movements but stayed around their original location, while short range and long range movements of POs constituted 30% and 10%, respectively. Comparably, most mitochondria were found to remain at a restricted location (75% static + 5% very short range movements), while only a small proportion performed short-range (5%) or long range movements (15%). When compared to mitochondria, we noted that POs performed significantly higher numbers of very short range movements, which to a high proportion represent small oscillating movements in the cytoplasm. While such movements are partially thermally-induced fluctuations, the high proportion of oscillating POs if compared to mitochondria might indicate that POs perform frequent coupling/uncoupling events to elements of the cytoskeleton. In contrast, many mitochondria in neurites might be more permanently attached to the cytoskeleton keeping them in a fixed position whereas POs might be constantly attaching and detaching to regulate PO motility.

Average velocities of all organelles analyzed were found to be 0.012 μm/sec for POs (Fig 2G) and 0.02 μm/sec for mitochondria (Fig 2H) corroborating that both organelles exhibit motilities in the same order of magnitude. However, as both organelles in the neurons rarely showed continuous movements for the whole analysis time, maximal actual velocities of organelles were significantly higher with 0.1 μm/sec for POs (Fig 2G) and 0.06 μm/sec for mitochondria (Fig 2H). These findings point to a similar transport mechanism applied to distribute both organelles in the cells. Indeed, Kif5 and Miro1 have been found to be involved in both PO and mitochondria transport via microtubules [32–34] suggesting a significant overlap in the molecular composition of their transport systems. In summary, POs and mitochondria in neurons appear to be generally rather static organelles, which only occasionally perform movements across long distances but appear to perform functions at rather stable positions inside the neurons, suggesting local mechanisms for metabolite exchange and organelle maintenance.

### Expression of the peroxisome-ER tether ACBD5 alter peroxisome motility in hippocampal neurons

**Fig 3:**
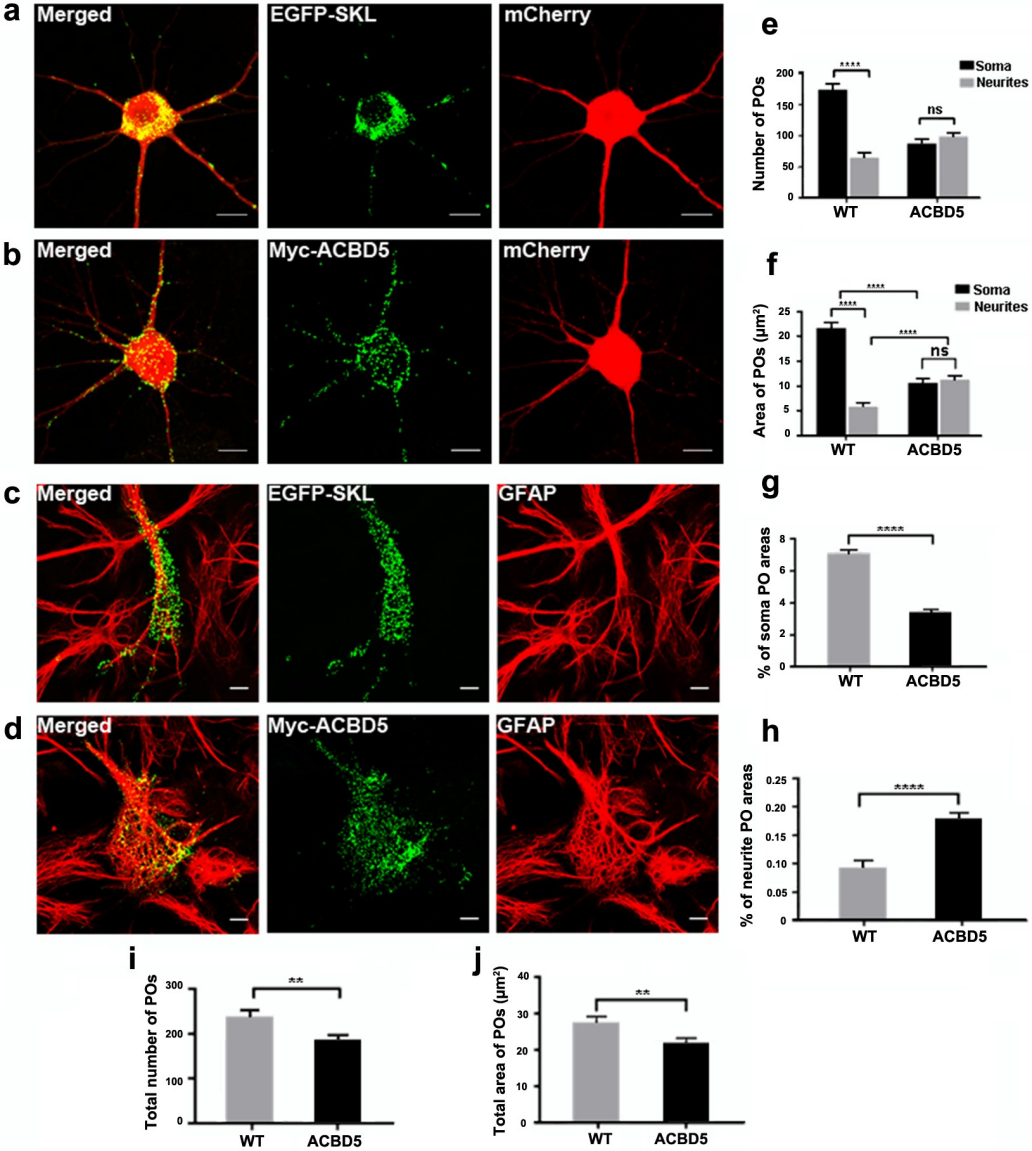
PO morphology and distribution in primary hippocampal neurons in response to the overexpression of myc-ACBD5. (a, b) POs in neurons co-transfected with plasmids encoding cytosolic mCherry as well as (a) EGFP-SKL and (b) myc-ACBD5 respectively. (c, d) PO distribution in astrocytes of the primary hippocampal cultures with (d) or without (c) myc-ACBD5 expression. Astrocytes were identified by GFAP immunostaining. Note that POs in astrocytes unlike in neurons are similarly distributed in both conditions. (e) PO numbers and (f) area distribution (μm^2^) in the soma and neurites (proximal 30 μm). (g, h) Percentage areas of POs in soma (g) and neurites (h) (proximal 30 μm). (i) Total PO number and (j) area covered by POs in transfected neurons. Representative immunofluorescence images are presented as maximum intensity projections (scale bar: 10 μm).

To investigate if the expression of the PO-ER tethering protein ACBD5 alters PO motility in neurons, primary hippocampal neurons at DIV7~9 were used for transfection experiments and analyzed by confocal live imaging 24 h after transfection. Differences in expression of individual plasmids could hamper a comparable PO identification in control and ACBD5 overexpressing test groups. To circumvent this problem, the hippocampal cultures were either transfected with plasmids encoding EGFP-SKL (Figs 2A and 3A) or myc-ACBD5 + EGFP-SKL (Fig 3B), respectively. The efficiency of double transfection using GFP-SKL and myc-ACBD5 encoding plasmids was controlled by fixation and immunostaining of the cultures after live imaging and was found to be ~80–90% in all cases. A plasmid coding for a mitochondria-targeted mCherry was co-transfected to monitor the mitochondrial motility as a reference (Fig 2A, red label). To evaluate if PO motilities are altered in response to ACBD5 expression, we applied automated tracking software, analyzing all POs in the neurites of a transfected neuron but excluding the soma. The respective graphs describe the empirical cumulative distribution function of the instantaneous PO speeds (Fig 2I) and covered distance (Fig 2L) for the wild type and ACBD5 expressing neurons. This shows the distribution of the total population of POs with each point of the curve corresponding to a single movement and indicates that a higher number of POs remain more static after ACBD5 expression. These findings are in line with the average velocities of the total PO population (Fig 2G, Average speed) as well as their average speed during the phases of movement (Fig 2G, Max speed). For a more detailed analysis, we again compared kymograph trajectories of POs and mitochondria in control and myc-ACBD5 expressing neurons and grouped the organelles into static organelles and those performing very short range, short range and long range movements as described above. In the myc-ACBD5 expressing cultures, the proportions of static and very short range moving POs increased from 1% to 3% and 59% to 81%, respectively, while those showing short and long range movements decreased from 30% to 14% and 10% to 2%, respectively (Fig 2E). Correspondingly, average speed (calculated across the whole analysis time) and average travelled distance of all analyzed POs decreased from 0.012 μm/sec to 0.0075 μm/sec (Fig 2G) and 5.6 μm to 3.6 μm in the myc-ACBD5 expressing neurons (Fig 2J), respectively. Of note, mitochondrial motility remained unchanged in response to ACBD5 expression (Fig 2F, 2H and 2K), demonstrating that the observed changes are PO-specific and do not result from a generally decreased fitness of the ACBD5 overexpressing cells.

Reduced average motilities and the reduction in long range movements in the total PO population might imply that the neuronal organelle transport system itself was compromised by the ACBD5 expression. Long range movements of POs in higher eukaryotes have been shown to be microtubule dependent [31, 32, 35]. When specifically long range movements are compared between the two groups, no significant differences were observed in either average or maximum speed of the moving POs (Fig 2N). Thus, according to our data, the mechanics of the microtubule transport does not change in response to ACBD5 expression but merely the number of motile POs decreases. Hence, POs which might interact with the ER via the ACBD5-VAPB tether could be efficiently prevented from entering the microtubule-based neuronal transport systems, whereas the kinesin/dynein-driven translocation mechanism itself appears to be unaltered in response to ACBD5 overexpression.

With respect to directionality of the transport, most PO movements did not result in a significant net anterograde or retrograde organelle translocation (Fig 2M). Within a time frame of 8 min, most mobile POs perform several back and forth movements along the axis of the neurites and exhibited net movement distances close to zero. In addition, the amount of POs with a net movement in an antero- and retrograde direction were comparable. These parameters did not significantly change in response to the myc-ACBD5 expression.

### ACBD5 expression redistributes peroxisomes into neurites and the periphery of perikarya

**Fig 4:**
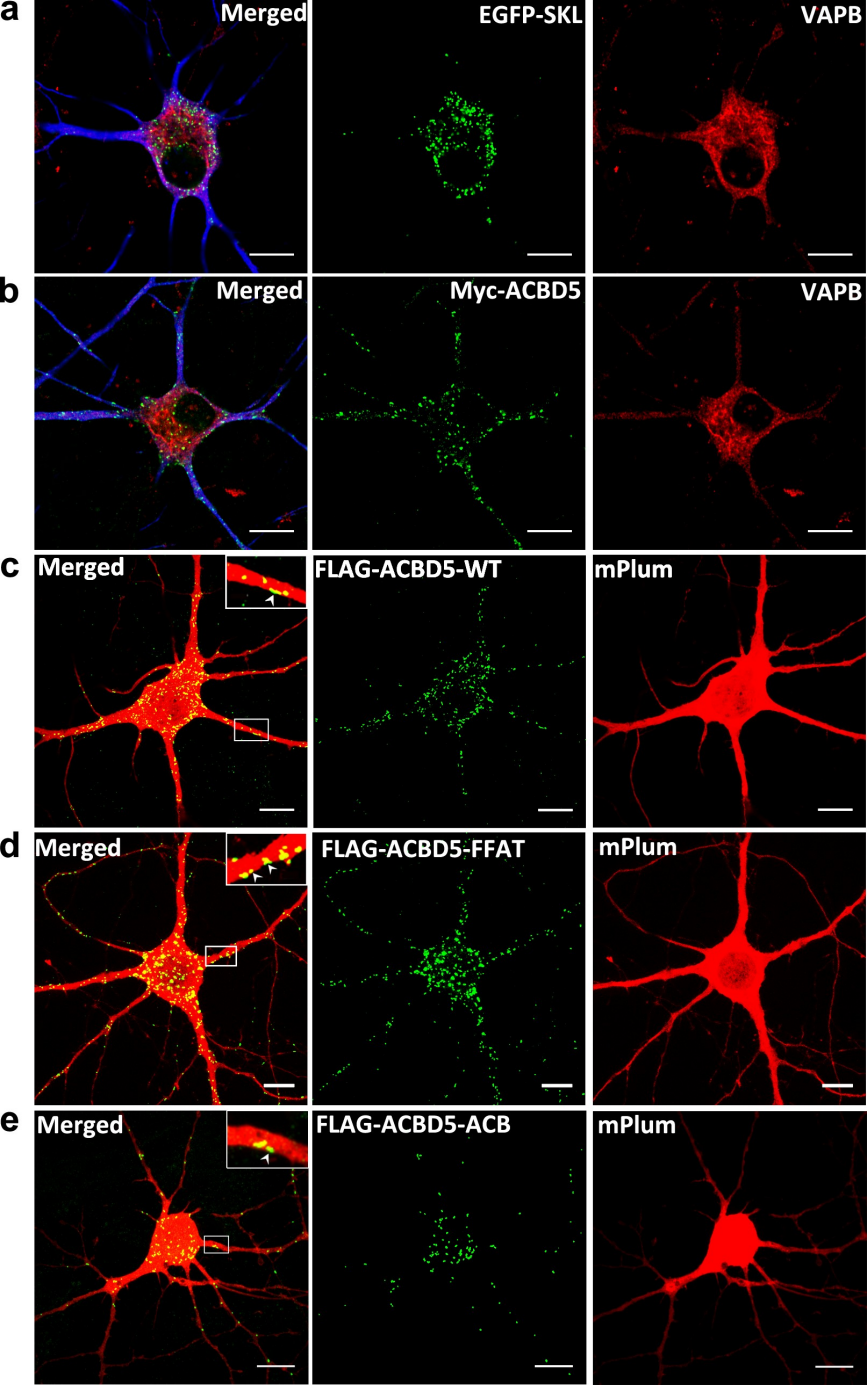
PO relocation in response to ACBD5 expression does not depend on the interaction with VAPB or acyl-CoA binding in neurons. (a, b) Localization of VAPB and (a) EGFP-SKL and (b) myc-ACBD5 in hippocampal neurons. MAP2-staining (in blue) was used as a neuronal dendrite marker (scale bar: 10 μm). (c, d) PO localization in neurons co-transfected with cytosolic mPlum-N1 and (c) FLAG-ACBD5-WT or (d) FLAG-ACBD5-FFAT or (e) FLAG-ACBD5-ACB expressing ACBD5 FFAT and ACB mutant proteins deficient in VAP or acyl-CoA binding, respectively. Both mutant proteins induce comparable PO relocations as compared to the wildtype ACBD5. Insets: Higher magnification of POs in close proximity to the plasma membrane (arrowheads) of dendrites (scale bar: 10 μm).

Since the expression of ACBD5 was found to interfere with the transport of POs along the neurites of the hippocampal neurons, we investigated if these changes in PO motility would have an effect on the distribution of POs in the neuron. Hippocampal neurons were transfected with plasmids encoding either EGFP-SKL or myc-ACBD, at DIV7~9 and fixed after 24 h. To identify the extended neurite compartment of the transfected neurons, a cytosolic mCherry was also co-expressed (Fig 3A and 3B). After immunostaining, we did not observe major alterations in PO morphology, which may interfere with efficient transport in neurons. Qualitatively, we observed that the POs in the soma were preferentially located at the cell periphery in the vicinity of the plasma membrane in the myc-ACBD5 group (Fig 3B). By comparison, in controls expressing EGFP-SKL, POs usually appeared in a juxtanuclear position (Fig 3A). To evaluate if these ACBD5-mediated PO rearrangements are exclusively found in neurons, we further analyzed the PO staining patterns in EGFP-SKL or myc-ACBD5 expressing astrocytes in the same hippocampal cultures. We found a minor tendency for an aggregation of POs in the cytoplasm, however, did not observe a trend towards a peripheral localization or an increase of POs in the astrocytic processes (Fig 3C and 3D). Moreover, in our previous study we did not observe such differences in PO distribution after ACBD5 expression in a variety of cell lines such as COS-7, HepG2 and human embryonic fibroblasts [36]. To quantify PO repositioning in neurons, the numbers and area of POs in the soma and in the surrounding neurite area (proximal 30 μm of each neurite) were compared. In the EGFP-SKL expressing control group, the average number of POs in the soma (n = 174) was far higher than in the proximal dendritic compartment (n = 65) (Fig 3E). The myc-ACBD5 expressing neurons, in contrast, exhibited more or less equal PO numbers in the soma (n = 88) and the analyzed proximal region of the neurites (n = 99). Since the relocation of POs towards the cellular periphery in response to myc-ACBD5 expression often resulted in POs closely apposed to each other, mere counting of POs could result in an underestimation of PO numbers. Therefore, the area of PO signals was used to directly compare changes in PO abundance in neurites and soma, respectively. While the area covered by POs in the soma decreased by a factor of 2 from 22 μm^2^ to 11 μm^2^, the area increased by a factor of 1.83 from 6 μm^2^ to 11 μm^2^ (Fig 3F). Taking into account that the neuron populations in the hippocampal culture are heterogeneous in size and shape, we further normalized the data to the analyzed soma and neurite area (Fig 3G and 3H). Comparison of percentage areas of POs in the soma and the neurites revealed that POs in the soma decreased from 7% in controls to 3% in response to myc-ACBD5 expression, while PO abundance doubled in the neurites (0.1% in controls, 0.2% in myc-ACBD5 expressing neurons) (Fig 3G and 3H). To evaluate if the differences of PO distribution were caused by the different PO markers myc-ACBD5 and EGFP-SKL, we additionally applied Pex14 antibody staining under both conditions, obtaining comparable differences in PO relocation (see S1 Fig). To exclude that the increased number of POs in the neurites may result from PO proliferation, we quantified the total number and area covered by POs in the total inspected neuron area (soma + proximal 30 μm of all neurites) (Fig 3I and 3J). Both values actually decreased after myc-ACBD5 expression, excluding such a possibility. Rather, the decreasing total number of POs in response to the expression of myc-ACBD5 is likely due to the fact that POs in the more distal parts of the neurites were not quantified in the analysis. Therefore, the most straightforward explanation for this PO reorganization would be that the POs are trapped at sites of ER subcompartments with a locally high VAPB concentration. To test such a hypothesis, primary hippocampal neurons were transfected with plasmids encoding EGFP-SKL and myc-ACBD5, respectively and then stained with an antibody against VAPB. As shown in Fig 4A and 4B, VAPB signals resemble a network-like staining pattern characteristic for the ER and concentrates in the somatic and the proximal dendritic area of the hippocampal neurons. In general, the staining is largely comparable to the signal pattern for an ER-marker plasmid like RFP-KDEL (Fig 1A, 1B and 1E), but revealed some particular focal concentrations, especially in the region surrounding the peri-nuclear Golgi apparatus. In line with our antibody staining, VAPB has been reported to facilitate vesicle trafficking at the ER-Golgi intermediate compartment (ERGIC) in rat hippocampal neurons [37]. Surprisingly, POs were not found to accumulate at sites with locally high VAPB concentrations but instead were found to juxtapose along the cell membrane in the soma and dendrites of the hippocampal neurons–areas which were largely devoid of VAPB signals (Fig 4B). Thus, the redistribution of POs in neurons appears to be cell-type specific. We concluded that the ACBD5-VAPB interaction connecting POs to the ER might not be responsible for the subcellular PO rearrangements described above. To substantiate this assumption, we expressed ACBD5 with a mutated FFAT (**2 ph**enylalanines in an **a**cidic **t**ract) motif. FFAT domains can be found in a variety of VAPB interacting proteins and are required for effective binding between VAPB and the interacting proteins [38]. Previously, we showed that this FFAT-mutant is unable to bind to VAPB using a pull-down approach (16). Furthermore, an ACBD5 construct with a mutated acyl-CoA binding domain (ACB) deficient in binding acyl-CoA esters [38] was used to analyze if changes in PO fatty acid metabolism might be responsible for the PO relocation. Thus, we compared the PO distribution of neurons transfected with plasmids coding for FLAG-ACBD5-FFAT, FLAG-ACBD5-WT and FLAG-ACBD5-ACB. To estimate if the constructs are expressed at comparable levels, immunoblots from COS-7 cells transfected with the 3 different plasmids variants were performed (the low transfection rates in the hippocampal cultures precluded a direct assessment of plasmid expression). As shown in S2 Fig, FLAG -ACBD5-WT and FLAG-ACBD5-FFAT exhibited largely comparable expression rates, while FLAG-ACBD5-ACB only reaches around 25% of the expression level of the WT construct. Remarkably, we observed that the POs, similar to WT ACBD5 expression (Fig 4C), distributed towards the plasma membrane and into the neurites of FLAG-ACBD5-FFAT as well as FLAG-ACBD5-ACB expressing neurons (Fig 4D and 4E). Interestingly, higher magnifications reveal that many POs in the dendrites can also be found in close proximity to the plasma membrane after expression of all ACBD5 variants (Figs 3B, 4C, 4D and 4E).

**Fig 5:**
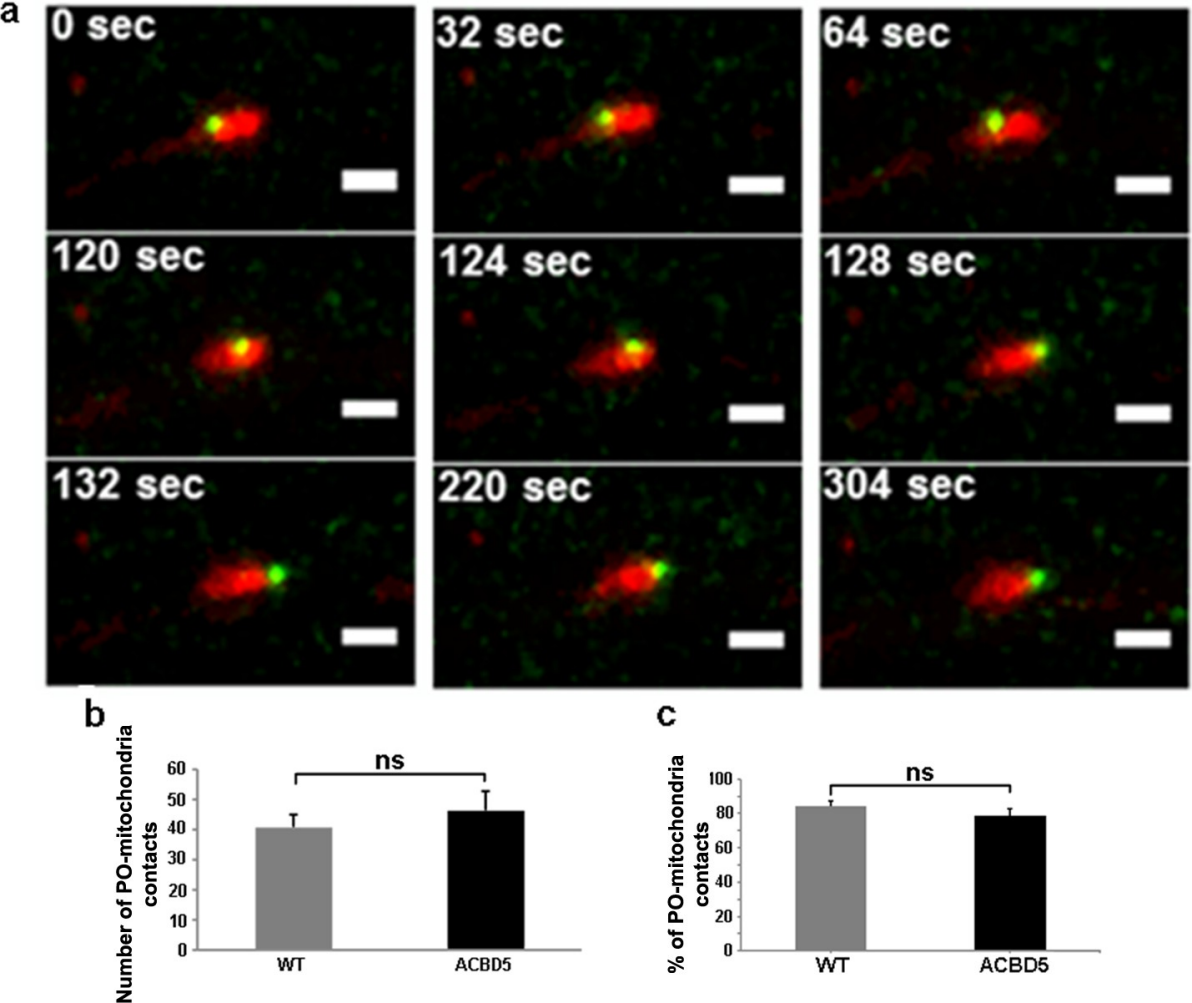
Close contacts of POs and mitochondria in live confocal images. (a) Time lapse series of a PO “surfing” on a mitochondrion over a period of 5 min (scale bar: 2 μm, maximum intensity projections). (b, c) Absolute numbers and proportion of POs juxtaposed to mitochondria in the neurite area of each hippocampal neuron.

During the organelle kinetic analysis we observed an unexpectedly high incidence of POs juxtaposed to mitochondria in the neurites of the hippocampal neurons (Fig 5). Moreover, some POs performed “surfing” movements along larger, static mitochondria, but did not migrate beyond the outline of the mitochondrion for the whole analysis time (Fig 5A) (S2 Movie). Similar PO movements along mitochondria have been described in the yeast species *Schizosaccharomyces pombe* [39]. Such a phenomenon may point to a functional interaction between both organelles, potentially associated with metabolite transfer or signaling events. Therefore, we asked if the PO repositioning in neurites in response to myc-ACBD5 expression might influence the interaction between POs and mitochondria. To this end, we quantified the proportion of POs in juxtaposition to mitochondria in the neurites of the hippocampal neurons of controls and after myc-ACBD5 expression. Using the live imaging data, we analyzed the spatial distribution of both organelles from the first frame of each dataset in the dendrites of the hippocampal neurons (Fig 5B and 5C). In controls, the percentage of POs located juxtaposed to mitochondria was found to be 84.6%—a remarkable value if compared to 20% as described for COS-7 cells [40]. Moreover, most POs remained stable at positions close to individual mitochondria. In myc-ACBD5 expressing neurons, we observed that the absolute number of POs juxtaposed to mitochondria was slightly increased. However, we found no significant change in the proportion of PO interacting with mitochondria (Fig 5C), indicating that the PO repositioning had no impact on the interaction between POs and mitochondria.

Taken together, we observed significant alterations in the motility and location of POs in response to overexpression of the molecular tether ACBD5. However, unlike in fibroblasts (16), interaction with the ER tethering protein VAPB as well as the protein’s acyl-CoA binding capacity was not found to be responsible for the observed relocation of POs. Rather our data indicates that ACBD5 might facilitate interactions with additional protein binding partners, which tether POs to the plasma membrane or unidentified organelles in its vicinity.

## Discussion

Organelle contact sites are increasingly regarded as important subcellular structures which permit the exchange of metabolites and signaling molecules between two or more subcellular compartments or even trigger organelle fission events [41]. Moreover, organelle contacts may also regulate positioning of individual organelles inside cells by counteracting loading of organelles to microtubule- or actin-based intracellular transport systems [42, 43]. In line with such a scenario, we recently observed that POs showed increased motility in human fibroblasts when PO-ER contacts were reduced [16]. By contrast, overexpression of a PO-targeted variant of the microtubule adaptor protein MIRO1 increased PO motility in COS-7 cells [44]. Thus, the extended network of ER tubules might act as an organellar scaffold, which retains vesicular organelles such as POs at defined intracellular locations counteracting the microtubule transport system. In that way, the network of ER tubules interacting with a variety of organelle types could act as a regulating factor in the maintenance of the subcellular cytoarchitecture. To investigate if ER tethering might alter PO distribution in the highly polarized neuronal cell type, we expressed the PO tethering factor ACBD5, which interacts with the ER protein VAPB to generate PO-ER contact sites [16, 17], in hippocampal primary cultures. The expression of ACBD5 decreased PO motilities in the dendritic compartment of neurons. In parallel, we observed conspicuous rearrangements in PO subcellular distribution: in the soma increasing amounts of POs were found in proximity to the cell membrane and an increased PO number was also observed in dendrites of transfected neurons. Unexpectedly, these subcellular rearrangements proved to be independent of the VAPB interaction as shown by transfection of an ACBD5-variant with a mutated FFAT domain, incapable of interacting with VAPB [16]. Expression of an acyl-CoA binding-deficient ACBD5 mutant further indicated, that the phenomenon is independent of the protein’s metabolic function. Thus, we hypothesize that ACBD5 interacts with other hitherto unidentified proteins which (a) might facilitate tethering to the plasma membrane/cortical ER or (b) might interfere with the connection of POs to the microtubule-based transport system as reported for PO redistribution in spastin mutant cells [45]. This latter possibility should preferentially alter fast, long range movements, which are usually microtubule-based, while slow, short range movements are usually actin-based [46]. However, according to our observations short and long range PO movements were similarly altered by ACBD5-expression, whereas very short range movements comprising largely of oscillating POs were increased. The latter behavior might indicate that POs destined to enter the microtubule transport system are permanently attaching to/detaching from microtubules since they are retained at a specific location by organelle tethering forces. Moreover, we did not observe any bias in retro- and anterograde PO transport in response to ACBD5 expression, which could explain the increased PO localization in the dendritic compartment of the hippocampal neurons. Since proper organelle distribution along the elongated dendritic and axonal processes is highly dependent on an efficient transport system, an immobilization of POs by organelle tethering would gradually slow down PO transport and consequently increase the number of POs in neurites. Thus, we conclude that organelle tethering might most likely be the molecular cause for the altered PO kinetics and redistribution in our hippocampal cultures, even if a specific interaction partner still has to be identified in future experiments.

Specifically, it remains to be determined why ACBD5 does not preferentially interact with VAPB as observed in other cell types [16, 17]. In addition to the FFAT binding motif, the ACBD5 amino acid sequence exhibits further predicted coiled-coil domains which are common protein interaction mediating structures [47]. Moreover, VAPB expression has been reported to be rather low in hippocampal neurons as compared to other neuronal cell types like motor neurons [48]. Thus, ACBD5, which according to RNA profiling data from the “Mouse ENCODE project” is expressed in significant amounts in the brain [49], might preferentially interact with another hitherto unidentified binding partner. In this regard it is tempting to speculate that ACBD5 might be a multivalent tethering factor, which can connect POs to distinct organelles in different cell types or in response to the physiologic state of a cell. Comparably, the mitochondrial membrane protein tyrosine phosphatase interacting protein 51 (PTPIP51), which also interacts with VAPB in an organelle tethering complex [50], has been reported to exhibit tissue-specific interactomes [51], thus indicating that PTPIP51 adjusts its protein interaction network to the specific functions of a cell type. Likewise the VAPA and VAPB proteins form tethering complexes with a plethora of different proteins connecting the ER to mitochondria, endosomes/lysosomes, the Golgi, peroxisomes, lipid droplets and the plasma membrane and thereby forms a flexible organelle interaction network [38]. ACBD5 might similarly act as a tethering factor which is able to trigger the formation of more than one organelle contact zone. Post-translational protein phosphorylation, which has been described for ACBD5 [52, 53], might serve to regulate the interaction with the different organelle-specific tethering complexes. It remains to be elucidated why POs might favor different organelle interactions in the hippocampal neurons. In addition to the ER, POs are known to specifically interact with mitochondria, for example at sites of acyl-CoA synthesis or mitochondria-ER junctions indicating distinct functional relationships at the contact zone [54]. In neurons, POs contribute to the maintenance of a defined redox potential inside the cell thereby influencing neuronal firing rates [9]. POs intricately cooperate with mitochondria in the regulation of cellular redox homeostasis [55]. The high incidence of PO-mitochondria contacts in the dendrites of hippocampal neurons observed in this study might be associated with the control of ROS generation; however, future studies are required to substantiate this hypothesis. In addition to the PO-mitochondria contacts not altered by myc-ACBD5 expression, we observed POs juxtaposed along the plasma membrane of the neurons which were more pronounced after ACBD5 expression. Recently, such contact sites have been described in yeast [56], implying that PO-plasma membrane associations might be common sites of organelle interaction. Functionally, PO localization in the vicinity of the plasma membrane might guarantee an efficient processing of plasma membrane lipids. POs along with lysosomes have been observed to be enriched in paranodal regions of myelin sheaths [57]. In Pex5^-/-^ mutants these lysosomes accumulated gangliosides from juxtaparanodal lipid rafts, which apparently could not be further metabolized in PO. This indicated complex interrelationships of local organelles in order to control local lipid composition of cellular membrane subcompartments [57].

In summary, this work is the first report documenting that changes in ACBD5 expression and correspondent PO-organelle interaction might regulate PO distribution in highly polarized neurons. Future studies are required to identify specific ACBD5-interaction partners which facilitate the redistribution of POs observed in this work. Moreover, there is currently a lack of information on the functional significance of locally confined PO populations inside neurons. Experimental manipulation of PO distribution in hippocampal primary cultures, as performed in this study, could be used as a model to analyze if proper organelle allocation influences the more specific electrophysiological neuronal functions.

## Supporting information

S1 Fig Analysis of PO distribution in primary hippocampal neurons in response to the overexpression of myc-ACBD5 by Pex14 immunostaining. (a, b) POs immunostained by Pex14 (green) in the neuronal cultures co-transfected with mPlum as a marker for the cytosol (red) as well as (a) EGFP-SKL and (b) myc-ACBD5, respectively. (c) PO numbers and (f) area distribution (μm^2^) in the soma and neurites (proximal 30 μm). (d) Total PO number and (g) area covered by POs in transfected neurons. (e, h) Ratios of PO number (e) and area (h) in neurites normalized to the total area of the neuron. Representative immunofluorescence images are presented as maximum intensity projections (scale bar: 10 μm).
(TIF)

S2 Fig Expression of ACBD5 variants in COS-7 cells. (a) Representative immunoblot showing the expression of the FLAG-WT-ACBD5, FLAG-FFAT-ACBD5 and FLAG-ACB-ACBD5, endogenous VAPB signals were used as internal loading control. (b) Relative signal intensities quantified from 3 independent experiments, pixel volumes for FLAG-WT-ACBD5 were set to 1.0.
(TIF)

S1 Movie Overview of 5 minutes of PO and mitochondria movements in DIV10 hippocampal neurons. POs and mitochondria were marked by EGFP-SKL and mPlum-Mito respectively.15 frames are displayed per second. Scale bar: 10μm. Note that most of the POs and mitochondria stay static in this overview.
(AVI)

S2 Movie Enlarged view of 8 minutes of POs and mitochondria movements in DIV10 hippocampal neurons. POs and mitochondria were marked by EGFP-SKL and mPlum-Mito respectively. 20 frames are displayed per second. Scale bar: 5 μm. Note that in the lower part a PO is “surfing” on a mitochondrion, implying a potential interaction.
(AVI)
