# Supplementary figures and images for: Intracellular redistribution of neuronal peroxisomes in response to ACBD5 expression
Source: PLoS One. 2018 Dec 27;13(12):e0209507. doi: 10.1371/journal.pone.0209507 (PMC6307868; doi:10.1371/journal.pone.0209507)

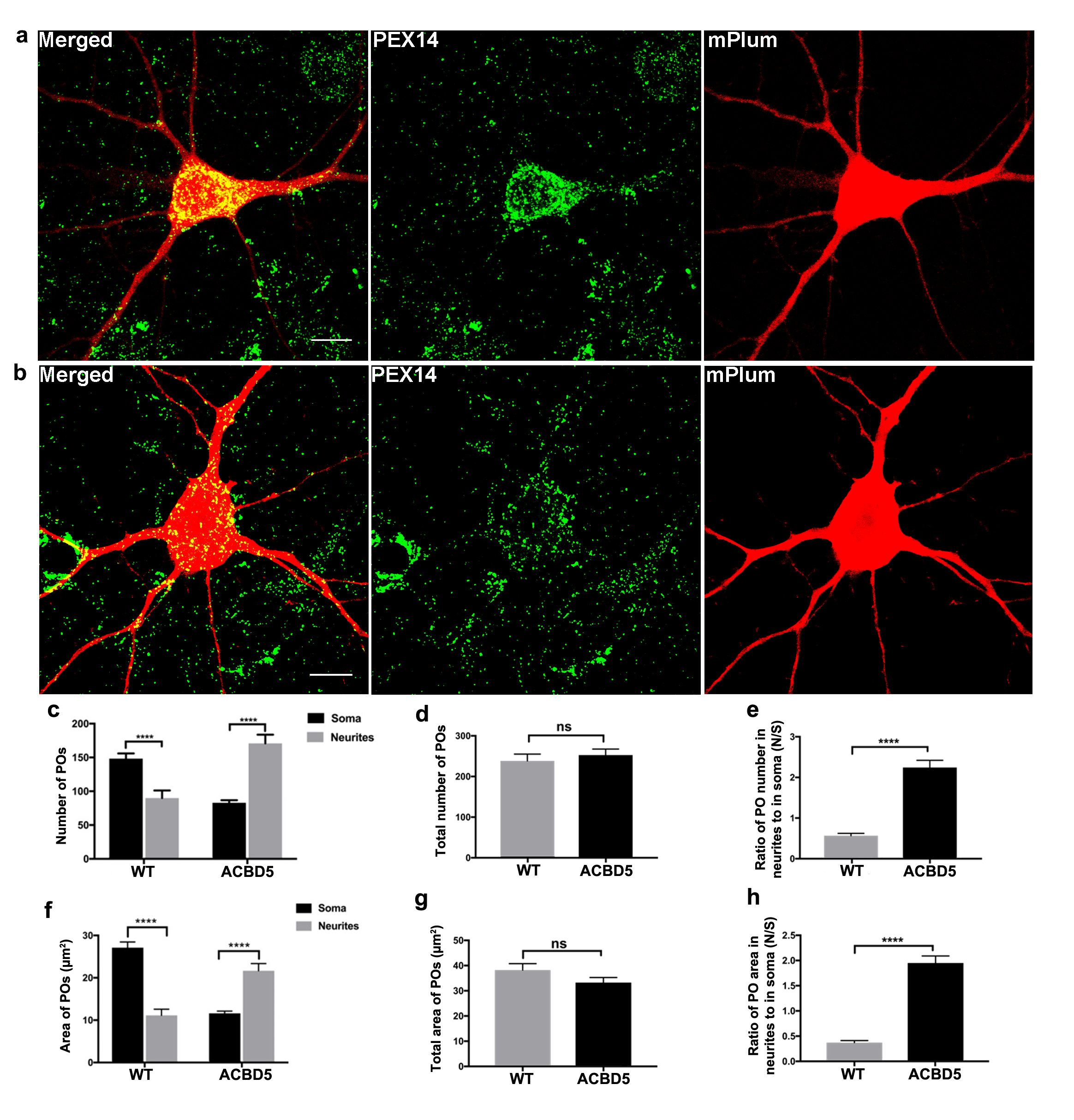

Supplement: S1 Fig — (a, b) POs immunostained by Pex14 (green) in the neuronal cultures co-transfected with mPlum as a marker for the cytosol (red) as well as (a) EGFP-SKL and (b) myc-ACBD5, respectively. (c) PO numbers and (f) area distribution (μm2) in the soma and neurites (proximal 30 μm). (d) Total PO number and (g) area covered by POs in transfected neurons. (e, h) Ratios of PO number (e) and area (h) in neurites normalized to the total area of the neuron. Representative immunofluorescence images are presented as maximum intensity projections (scale bar: 10 μm). (TIF) [file pone.0209507.s001.tif]

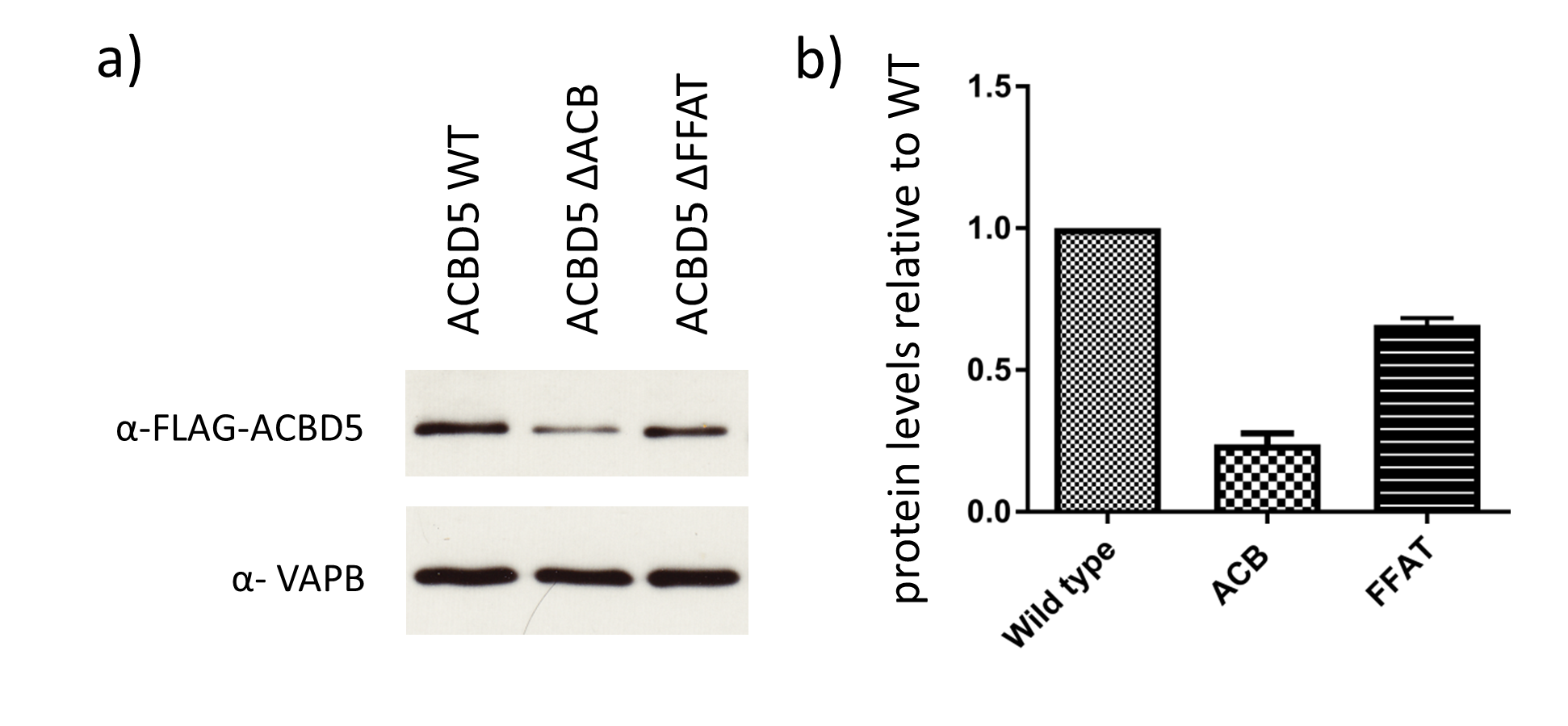

Supplement: S2 Fig — (a) Representative immunoblot showing the expression of the FLAG-WT-ACBD5, FLAG-FFAT-ACBD5 and FLAG-ACB-ACBD5, endogenous VAPB signals were used as internal loading control. (b) Relative signal intensities quantified from 3 independent experiments, pixel volumes for FLAG-WT-ACBD5 were set to 1.0. (TIF) [file pone.0209507.s002.tif]
